# Xenohormetic, hormetic and cytostatic selective forces driving longevity at the ecosystemic level

**DOI:** 10.18632/aging.100186

**Published:** 2010-08-07

**Authors:** Alexander A. Goldberg, Pavlo Kyryakov, Simon D. Bourque, Vladimir I. Titorenko

**Affiliations:** Department of Biology, Concordia University, Montreal, Quebec H4B 1R6, Canada

**Keywords:** Aging, longevity, evolution, ecosystems, hormesis, xenohormesis, link between growth and aging, quasi-programmed aging, anti-aging compounds, resveratrol, rapamycin, bile acids

## Abstract

We recently found that lithocholic acid (LCA), a bile acid, extends yeast longevity. Unlike mammals, yeast do not synthesize bile acids. We therefore propose that bile acids released into the environment by mammals may act as interspecies chemical signals providing longevity benefits to yeast and, perhaps, other species within an ecosystem.

## Bile acids delay aging in yeast via two different mechanisms

**Figure 1.**
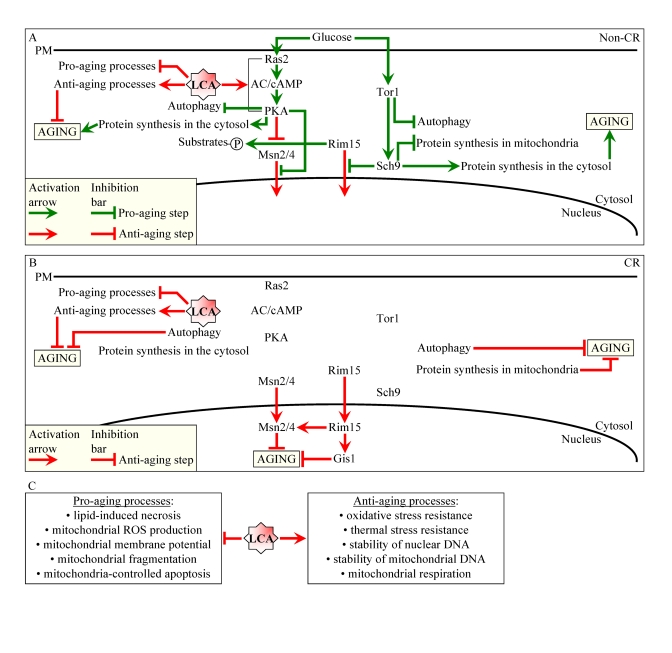
Lithocholic acid (LCA) extends longevity of chronologically aging yeast through two different mechanisms. (**A** and **B**) Outline of pro- and anti-aging processes that are controlled by the TOR and/or cAMP/PKA signaling pathways and are modulated by LCA in yeast cells grown under non-CR (**A**) or CR (**B**) conditions. Activation arrows and inhibition bars denote pro-aging (displayed in green color) or anti-aging (displayed in red color) processes. Under both non-CR and CR conditions, LCA targets housekeeping longevity assurance processes listed in (**C**). Under non-CR conditions only, LCA also targets the adaptable cAMP/PKA pathway. By activating PKA-dependent phosphorylation of the cytosolic pool of the key nutrient-sensory protein kinase Rim15p, LCA causes the inactivation of Rim15p. The resulting reduction of the phosphorylation status of several Rim15p target proteins in the cytosol lowers their pro-aging efficacy. Abbreviations: CR, caloric restriction; PM, plasma membrane.

We recently found that LCA greatly (and some other bile acids to a lesser degree) increases the chronological life span of yeast under caloric restriction (CR) conditions [1]. Our findings provided evidence that LCA extends longevity of chronologically aging yeast through two different mechanisms (Figure 1).

 In one mechanism, this bile acid targets longevity pathways that control chronological aging irrespective of the number of calories available to yeast. Because these pathways modulate longevity regardless of calorie availability, we called them "constitutive" or "housekeeping" [1]. LCA modulates these housekeeping longevity assurance pathways by suppressing lipid-induced necrosis, attenuating mitochondrial fragmentation, altering oxidation-reduction processes in mitochondria, enhancing resistance to oxidative and thermal stresses, suppressing mitochondria-controlled apoptosis, and enhancing stability of nuclear and mitochondrial DNA ([1]; Figure 1C). The housekeeping longevity pathways do not overlap with the TOR (target of rapamycin) and cAMP/PKA (cAMP/protein kinase A) signaling pathways ([1]; Figure 1A), both of which are "adaptable"  by nature because they are under the stringent control of calorie and/or nutrient availability ([2-6]; Figure 1B).

 In the other mechanism, LCA targets the adaptable cAMP/PKA pathway by unmasking an anti-aging potential of PKA under non-CR conditions, perhaps by activating PKA-dependent phosphorylation of the cytosolic pool of the key nutrient-sensory protein kinase Rim15p [1]. The phosphorylation of Rim15p by PKA inactivates its protein kinase activity [7]. Hence, the LCA-driven inactivation of Rim15p may reduce the phosphorylation status of its known [8] target proteins in the cytosol, thereby lowering their pro-aging efficacy ([1]; Figure 1A).

## Bile acids are beneficial to health and longevity in animals

**Figure 2.**
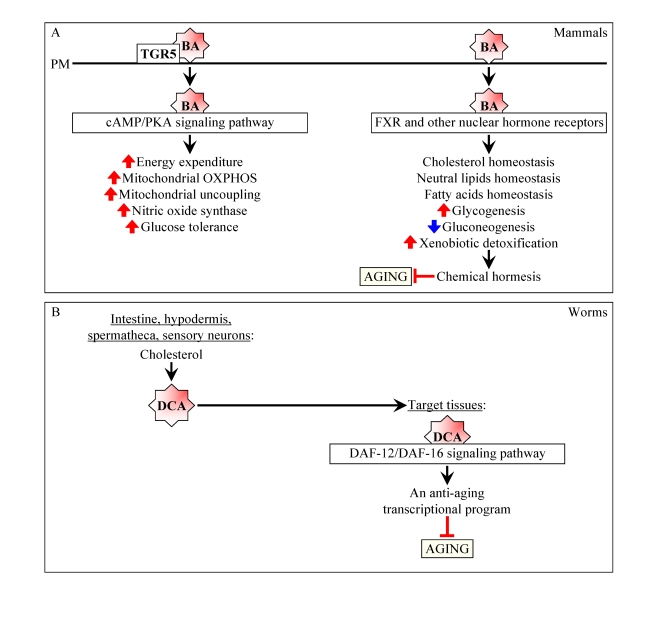
Bile acids are beneficial to health and longevity in animals. (**A**) In mammals, bile acids (BA) function not only as trophic factors for the enteric epithelium and detergents for the emulsification and absorption of dietary lipids, but also as signaling molecules that regulate lipid, glucose and energy homeostasis and activate detoxification of xenobiotics.By improving overall health, BA may delay the onset of age-related diseases and have beneficial effect on longevity. By activating transcription of numerous xenobiotic detoxification genes and thus promoting chemical hormesis, BA may extend their longevity by acting as endobiotic regulators of aging. (**B**) In worms, following their synthesis from cholesterol in the intestine, hypodermis, spermatheca and sensory neurons, bile acid-like dafachronic acids (DCA) are delivered to other tissues where they activate the DAF-12/DAF-16 signaling cascade, thereby orchestrating an anti-aging transcriptional program and increasing the life span of the entire organism.

Although bile acids in mammals have been traditionally considered only as trophic factors for the enteric epithelium and detergents for the emulsification and absorption of dietary lipids [9-11], they are now also recognized for their essential role as signaling molecules regulating lipid, glucose and energy homeostasis and activating detoxification of xenobiotics ([9-14]; Figure 2A). Many of the numerous health-improving metabolic effects caused by bile acids and their demonstrated ability to protect mammals from xenobiotic toxins ([9-14]; Figure 2A) suggest that, by improving overall health, these amphipathic molecules may delay the onset of age-related diseases and have beneficial effect on longevity. Furthermore, because of the elevated levels of several bile acids in the long-lived Ghrhr^lit/lit^ mice and due to the ability of cholic acid administered to food of wild-type mice to activate transcription of numerous xenobiotic detoxification genes, it has been proposed that, by promoting chemical hormesis in mammals, these mildly toxic molecules with detergent-like properties may extend their longevity by acting as endobiotic regulators of aging [15-18]. Moreover, bile acid-like dafachronic acids (including 3-keto-LCA) in worms function as endocrine regulators of aging by activating an anti-aging transcriptional program governed by the DAF-12/DAF-16 signaling cascade ([19-21]; Figure 2B). Altogether, these findings support the notion that bile acids are beneficial to health and longevity in animals because of their ability to operate as potent signaling molecules that modulate a compendium health- and longevity-related processes. Noteworthy, by modulating many of these processes also in yeast, LCA extends their longevity [1]. It is likely therefore that the life-extending capacity of LCA and other bile acids as well as, perhaps, the mechanisms underlying their anti-aging action are conserved across animal species and other phyla.

## Bile acids may function as interspecies chemical signals extending yeast longevity within ecosystems

Importantly, yeast do not synthesize LCA or any other bile acid found in mammals [1,11,22]. We therefore hypothesize that bile acids released into the environment by mammals may act as interspecies chemical signals providing longevity benefits to yeast. In our hypothesis, these mildly toxic compounds released into the environment by mammals may create selective pressure for the evolution of yeast species that can respond to the resulting mild cellular damage by developing the most efficient stress protective mechanisms. Such mechanisms may provide effective protection of yeast not only against cellular damage caused by bile acids (and, perhaps, by other environmental xenobiotics) but also against molecular and cellular damage accumulated with age. In our hypothesis, yeast species that have been selected for the most effective mechanisms providing protection against bile acids (and other environmental xenobiotics) are expected to evolve the most effective anti-aging mechanisms that are sensitive to regulation by bile acids (and, perhaps, by other environmental xenobiotics). Thus, the ability of yeast to sense bile acids produced by mammals and then to respond by undergoing certain life-extending changes to their physiology (Figure 1) is expected to increase their chances of survival, thereby creating selective force aimed at maintaining such ability.

## Natural variations of bile acid levels within ecosystems may modulate both housekeeping and adaptable longevity pathways in yeast

Noteworthy, the bulk quantity of bile acids in mammals exists as an organismal pool which cycles between intestine and liver in the enterohepatic circulation due to the efficient reabsorption of bile acids in the terminal ileum [10,11]. However, about 5% (up to 600 mg/day) of this pool escapes each reabsorption cycle, being continuously released into the large intestine and ultimately into the environment [10,11]. Thus, yeast are permanently exposed to bile acids due to their fecal loss by mammals. It is conceivable therefore that, in yeast exposed to bile acids released by mammals, these interspecies chemical signals modulate housekeeping longevity assurance pathways that 1) regulate yeast longevity irrespective of the state of the environment or food supply (*i.e*., the number of available calories and nutrients); and 2) do not overlap (or only partially overlap) with the adaptable TOR and cAMP/PKA longevity pathways that are under the stringent control of calorie and nutrient availability.

 It should be stressed, however, that the quantity of bile acids released into the environment by mammals could vary due to changes in the density of mammalian population and, perhaps, due to other environmental factors (including the abundance of food available to mammals, its nutrient and caloric content, and its fat mass and quality). In fact, the organismal pool of bile acids in mammals is under the stringent control of regulatory mechanisms operating in the liver during the fasting-refeeding transition [9-11]. Hence, it is likely that, in addition to the ability of yeast to respond to the permanently available exogenous pool of bile acids by modulating some housekeeping longevity assurance pathways, they have also evolved the ability to sense the environmental status-dependent variations of bile acids abundance by modulating the adaptable TOR and cAMP/PKA longevity pathways. Importantly, our recent study provided evidence for two mechanisms underlying the life-extending effect of LCA in yeast; one mechanism involves the calorie supply-independent modulation of a compendium of housekeeping longevity assurance processes that are not regulated by the TOR and cAMP/PKA pathways, whereas the other mechanism operates only in yeast on a calorie-rich diet by unmasking the previously unknown anti-aging potential of the calorie supply-dependent PKA [1].

It remains to be seen if our hypothesis on the essential role of bile acids as interspecies chemical signals regulating longevity in yeast is applicable to other species routinely exposed to bile acids within an ecosystem, such as plants and bacteria.

## Rapamycin may also act as an interspecies chemical signal modulating longevity at the ecosystemic level

Our hypothesis on longevity regulation by bile acids within ecosystems may explain the evolutionary origin of the life-extending effect of another anti-aging compound, called rapamycin. Synthesized by soil bacteria to inhibit growth of fungal competitors, this macrocyclic lactone provides longevity benefit to yeast, fruit flies and mice by specifically inhibiting TOR (Tor1p in yeast), a nutrient-sensory protein kinase that operates as a master negative regulator of the key adaptable longevity pathway [3,4,23-25]. Because rapamycin delays proliferative growth of organisms across phyla by causing G1 cell cycle arrest [3,4,26], it could be considered as a mildly cytotoxic compound, akin to bile acids (Our recent unpublished data revealed that rapamycin is a more toxic hormetic molecule than LCA and other bile acids). We propose therefore that, following its release into the environment by soil bacteria, rapamycin may create selective pressure for the evolution of yeast, fly and mammalian species that can respond to rapamycin-induced growth retardation by developing certain mechanisms aimed at such remodeling of their anabolic and catabolic processes that would increase their chances of survival under conditions of slow growth. It is plausible that some of these mechanisms delay aging by optimizing essential longevity-related processes and remain sensitive to modulation by rapamycin. Hence, the ability of yeast, fruit flies and mice to sense rapamycin produced by soil bacteria and then to respond by undergoing certain life-extending changes to their physiology is expected to increase their chances of survival, thereby creating selective force for maintaining such ability.

Interestingly, rapamycin has been shown to increase life span in fruit flies under dietary restriction conditions [25], when the TOR-governed adaptable pro-aging pathways are fully suppressed and the TOR-governed adaptable anti-aging pathways are fully activated [3,4]. It is plausible therefore that - similar to the proposed above anti-aging mechanism of LCA in yeast - rapamycin in fruit flies can modulate both the housekeeping (TOR-independent) and adaptable (TOR-dependent) longevity pathways. Hence, it is tempting to speculate that, in addition to the ability of fruit flies to respond to the permanently available exogenous pool of rapamycin by modulating some housekeeping longevity assurance pathways, they have also evolved the ability to sense the environmental status-dependent variations of rapamycin abundance (due to, *e.g.*, changes in the density of soil bacteria population) by modulating the TOR-governed adaptable longevity pathways. Of note, recent findings in yeast imply that - in addition to its role as a master negative regulator of the key adaptable longevity pathway - Tor1p may also operate as a positive longevity regulator, in particular by stimulating nuclear import of the transcriptional factors Sfp1p, Rtg1 and Rtg3 in response to partial mitochondrial dysfunction or changes in the exogenous and endogenous levels of glutamate and glutamine [27-29]. The ability of these transcriptional factors to regulate metabolism, ribosome biogenesis and growth is crucial for longevity [28,30,31].

## The "xenohormesis" hypothesis: a case of xenohormetic phytochemicals

Our hypothesis on longevity regulation by bile acids and rapamycin within ecosystems complements the "xenohormesis" hypothesis, in which plants and other autotrophic organisms respond to various environmental stresses (*i.e.*, UV light, dehydration, infection, predation, cellular damage and nutrient deprivation) by synthesizing a compendium of secondary metabolites [32-34]. Within plants and other autotrophs producing these phytochemicals in response to environmental stresses, they activate defense systems protecting the host organisms against such stresses. In addition, these phytochemicals constitute a chemical signature of the environmental status of an ecosystem. As such, they provide to heterotrophic organisms (*i.e*., animals and fungi) within the ecosystem an advance warning about deteriorating environmental conditions [33]. By operating as interspecies chemical signals, they could create selective pressure for the evolution of heterotrophic organisms that can sense these signals and then to respond by altering their metabolism in defensive preparation for the imminent adversity while conditions are still favorable. The resulting metabolic remodeling causes such specific changes in physiology of heterotrophs that are beneficial to their health and longevity [33]. Although xenohormetic phytochemicals are produced by autotrophic organisms only in response to hormetic environmental stresses, it is unlikely that they function as mildly toxic hormetic molecules within heterotrophic organisms; rather, the xenohormesis hypothesis proposes that the beneficial to health and longevity effects of xenohormetic phytochemicals are due to their well known ability to modulate the key enzymes of stress-response pathways governing numerous longevity-related processes in heterotrophic organisms [33-42]. The xenohormetic mode of positive selection for the most efficient longevity regulation mechanisms has been proposed to be driven by such phytochemicals as resveratrol, butein, fisetin and other polyphenols, as well as by curcumin [32-34]. The ability of caffeine to increase yeast chronological life span by decreasing the catalytic activity of Tor1p [43] suggests that this xanthine alkaloid could also operate as a xenohormetic phytochemical signal providing an advance warning about deteriorating environmental conditions to yeast, thereby driving the evolution of their longevity regulation mechanisms.

**Figure 3.**
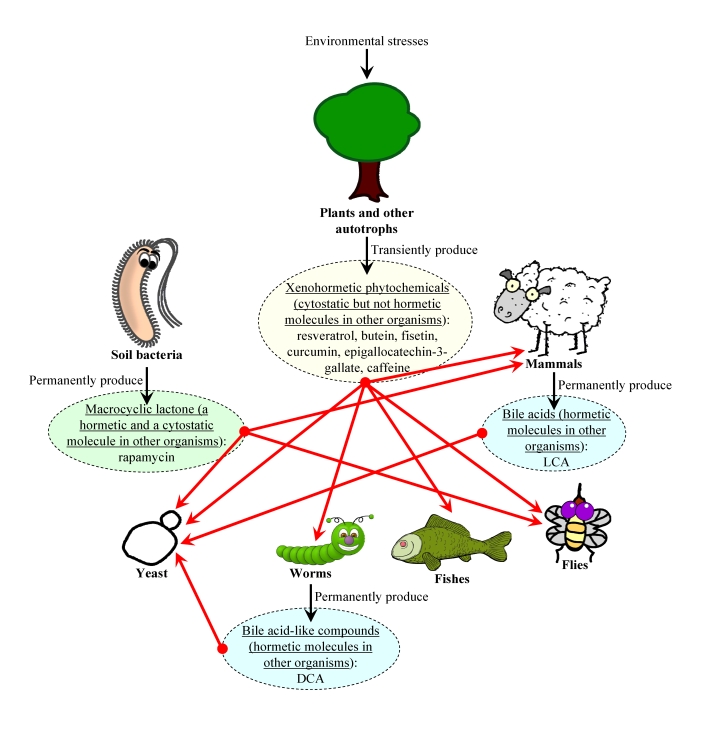
The xenohormetic, hormetic and cytostatic selective forces may drive the evolution of longevity regulation mechanisms within an ecosystem. We propose that organisms from all domains of life within an ecosystem synthesize chemical compounds that 1) are produced and then released into the environment permanently or only in response to deteriorating environmental conditions, increased population density of competitors and/or predators, or changes in food availability and its nutrient and/or caloric content; 2) are mildly toxic compounds that trigger a hormetic response in an organism that senses them or, alternatively, are not toxic for any organism within the ecosystem and do not cause a hormetic response; 3) are cytostatic compounds that attenuate the TOR-governed signaling network or, alternatively, do not modulate this growth-promoting network; and 4) extend longevity of organisms that can sense these compounds (red arrows), thereby increasing their chances of survival and creating selective force aimed at maintaining the ability of organisms composing the ecosystem to respond to these compounds by undergoing specific life-extending changes to their physiology. In our hypothesis, the evolution of longevity regulation mechanisms in each group of the organisms composing an ecosystem is driven by the ability of this group of organisms to undergo specific life-extending changes to their physiology in response to a compendium of "critical" chemical compounds that are permanently or transiently released to the ecosystem by other groups of organisms. Abbreviations: LCA, lithocholic acid; DCA, bile acid-like dafachronic acids.

## The "anti-aging side effect" hypothesis: delaying aging by attenuating the growth-promoting TOR signaling pathway

A common feature of many anti-aging compounds - some of which are mildly toxic hormetic molecules, whereas the others are non-toxic xenohormetic phytochemicals - is that they exhibit a cytostatic effect by inhibiting TOR, a nutrient-sensing signaling pathway that promotes proliferative growth in all heterotrophic organisms. A recently proposed "anti-aging side effect" hypothesis envisions that the primary objective for the synthesis of these cytostatic compounds by a group of the organisms composing an ecosystem is to suppress growth of other group(s) of organisms within this ecosystem, thereby killing competitors and/or protecting themselves from predators [39]. Due to its central role in promoting proliferative growth of all heterotrophic organisms, the TOR signaling pathway is a preferable target of such cytostatic compounds [3,26,39,44, 45]. Because the TOR pathway provides a molecular link between growth and aging by driving a so-called quasi-programmed aging [3,44,45], these compounds exhibit a side effect of suppressing aging [39]. In fact, soil bacteria synthesize rapamycin to suppress growth of fungal competitors by inhibiting the TOR protein kinase, a master positive regulator of the TOR signaling pathway that drives developmental growth of young organisms [3,23-25]. However, since - according to the anti-aging side effect hypothesis - in heterotrophic organisms across phyla this pathway also drives aging after their developmental growth is completed [44,45], rapamycin has a side effect of suppressing aging of all groups of heterotrophic organisms within an ecosystem [39]. Moreover, the anti-aging side effect hypothesis predicts that plants synthesize resveratrol in part to protect their grapes by inhibiting fungal growth [39]. Yet, because this small polyphenol attenuates the TOR signaling pathway by modulating key upstream regulators and downstream targets of the TOR protein kinase [35-42], resveratrol also displays a side effect of slowing down quasi-programmed TOR-driven aging of various species of heterotrophic organisms within an ecosystem [39].

In the anti-aging side effect hypothesis, cytostatic compounds attenuating the TOR pathway operate as interspecies chemical signals that provide longevity benefits to a range of heterotrophic organisms composing an ecosystem [39]. We propose that, following their release into the environment by soil bacteria or plants, these growth suppressing chemical compounds may create selective pressure for the evolution of yeast, worm, fly and mammalian species that can respond to the resulting retardation of their growth by developing certain mechanisms aimed at specific remodeling of the TOR-governed signaling network. By targeting the TOR protein kinase itself and/or its numerous upstream regulators and downstream targets, such mechanisms may attenuate the hyper-activation of TOR-governed cellular signaling pathways and cellular functions that - according to the concept of quasi-programmed TOR-driven aging [44,45] - are initiated after developmental growth of a heterotrophic organism is completed. In our hypothesis, the species of heterotrophic organisms that have been selected for the most efficient mechanisms preventing the hyper-activation of TOR-governed cellular signaling pathways and cellular functions following the completion of developmental growth are expected to evolve the most effective anti-aging mechanisms. Such mechanisms may be sensitive to various environmental factors, including the density of organism population and abundance of nutrients within an ecosystem.

## The xenohormetic, hormetic and cytostatic selective forces may drive the evolution of longevity regulation mechanisms within ecosystems

Unlike xenohormetic phytochemicals that are non-toxic compounds transiently synthesized and released by autotrophs only in response to environmental stresses [33,34], bile acids are mildly toxic hormetic molecules that are permanently synthesized and released by mammals [9-11,14-18]. Furthermore, rapamycin is a more toxic hormetic molecule than bile acids (our unpublished data) that is permanently synthesized and released by soil bacteria [46]. Moreover, many xenohormetic phytochemicals and mildly toxic hormetic molecules exhibit a cytostatic effect by attenuating TOR-governed cellular signaling pathways and cellular functions [39]. Therefore, by fusing the xenohormesis hypothesis [32-34], the anti-aging side effect hypothesis [39] and the proposed here hypothesis on longevity regulation by bile acids and rapamycin within ecosystems, we put forward a unified hypothesis of the xenohormetic, hormetic and cytostatic selective forces driving the evolution of longevity regulation mechanisms at the ecosystemic level.

 In our unified hypothesis (Figure 3), organisms from all domains of life (*i.e.*, bacteria, fungi, plants and animals) within an ecosystem are able to synthesize chemical compounds that 1) are produced and then released into the environment permanently or only in response to deteriorating environmental conditions, increased population density of competitors and/or predators, or changes in food availability and its nutrient and/or caloric content; 2) are mildly toxic compounds that trigger a hormetic response in an organism that senses them or, alternatively, are not toxic for any organism within the ecosystem and do not cause a hormetic response; 3) are cytostatic compounds that attenuate the TOR-governed signaling network (*e.g*., rapamycin and resveratrol) or, alternatively, do not modulate this growth-promoting network (*e.g*., LCA and other bile acid) and 4) extend longevity of organisms that can sense these compounds, thereby increasing their chances of survival and creating selective force aimed at maintaining the ability of organisms composing the ecosystem to respond to these compounds by undergoing specific life-extending changes to their physiology. Our hypothesis implies that the evolution of longevity regulation mechanisms in each group of the organisms composing an ecosystem is driven by the ability of this group of organisms to undergo specific life-extending physiological changes in response to a compendium of "critical" chemical compounds that are permanently or transiently released to the ecosystem by other groups of organisms.

## Verification of our hypothesis

As the first step towards testing the validity of our hypothesis of the xenohormetic, hormetic and cytostatic selective forces driving the evolution of longevity regulation mechanisms within ecosystems, we are currently carrying out the LCA-driven experimental evolution of longevity regulation mechanisms in chronologically aging yeast cultured under laboratory conditions. If we could select long-lived yeast species following a long-term exposure of wild-type yeast to LCA, we would be able to begin addressing the following intriguing questions: 1) what genes are affected by mutations responsible for the extended longevity of selected long-lived yeast species? 2) how these mutations influence a compendium of the housekeeping longevity-related processes modulated by LCA in chronologically aging yeast ([1]; Figure 1); 3) will these mutations affect the growth rate of yeast in media with or without LCA? 4) will selected long-lived yeast species be able to maintain their ability to live longer than wild-type yeast if they undergo several successive passages in medium without LCA? - and, thus, is there selective pressure aimed at maintaining of an "optimal" rather than a "maximal" chronological life span of yeast (due to, *e.g.*, a proposed selective advantage of the envisioned "altruistic" program [47-52] of chronological aging in yeast)? and 5) if mixed with an equal number of wild-type yeast cells, will selected long-lived yeast species out-grow and/or out-live them in medium without LCA or the opposite will happen (due to selective pressure on yeast aimed at maintaining of the so-called "altruistic" program [47-52] of their chronological aging)?
